# Gene expression and molecular pathway activation signatures of *MYCN*-amplified neuroblastomas

**DOI:** 10.18632/oncotarget.19662

**Published:** 2017-07-28

**Authors:** Ivan Petrov, Maria Suntsova, Elena Ilnitskaya, Sergey Roumiantsev, Maxim Sorokin, Andrew Garazha, Pavel Spirin, Timofey Lebedev, Nurshat Gaifullin, Sergey Larin, Olga Kovalchuk, Dmitry Konovalov, Vladimir Prassolov, Alexander Roumiantsev, Anton Buzdin

**Affiliations:** ^1^ D. Rogachev Federal Research Center of Pediatric Hematology, Oncology and Immunology, Moscow, Russia; ^2^ First Oncology Research and Advisory Center, Moscow, Russia; ^3^ Moscow Institute of Physics and Technology, Moscow, Russia; ^4^ V.A. Trapeznikov Institute of Control Sciences, Russian Academy of Sciences, Moscow, Russia; ^5^ Group for Genomic Regulation of Cell Signaling Systems, Shemyakin-Ovchinnikov Institute of Bioorganic Chemistry, Moscow, Russia; ^6^ Department of Oncology, Hematology and Radiology, N.I.Pirogov Russian National Research Medical University, Moscow, Russia; ^7^ National Research Centre “Kurchatov Institute”, Centre for Convergence of Nano-, Bio-, Information and Cognitive Sciences and Technologies, Moscow, Russia; ^8^ Pathway Pharmaceuticals, Hong Kong, China; ^9^ Centre for Biogerontology and Regenerative Medicine, IC Skolkovo, Moscow, Russia; ^10^ Engelhardt Institute of Molecular Biology, Russian Academy of Sciences, Mosow, Russia; ^11^ Moscow State University, Faculty of Fundamental Medicine, Moscow, Russia; ^12^ Department of Biological Sciences, University of Lethbridge, Lethbridge, Canada; ^13^ Federal State Budgetary Educational Institution of Further Professional Education “Russian Medical Academy of Continuous Professional Education” of the Ministry of Healthcare of the Russian Federation, Moscow, Russia

**Keywords:** neuroblastoma, MYCN-amplification, pediatric, gene expression, signaling pathways

## Abstract

Neuroblastoma is a pediatric cancer arising from sympathetic nervous system. Remarkable heterogeneity in outcomes is one of its widely known features. One of the traits strongly associated with the unfavorable subtype is the amplification of oncogene *MYCN*. Here, we performed cross-platform biomarker detection by comparing gene expression and pathway activation patterns from the two literature reports and from our experimental dataset, combining profiles for the 761 neuroblastoma patients with known *MYCN* amplification status. We identified 109 / 25 gene expression / pathway activation biomarkers strongly linked with the *MYCN* amplification. The marker genes/pathways are involved in the processes of purine nucleotide biosynthesis, ATP-binding, tetrahydrofolate metabolism, building mitochondrial matrix, biosynthesis of amino acids, tRNA aminoacylation and NADP-linked oxidation-reduction processes, as well as in the tyrosine phosphatase activity, p53 signaling, cell cycle progression and the G1/S and G2/M checkpoints. To connect molecular functions of the genes involved in *MYCN*-amplified phenotype, we built a new molecular pathway using known intracellular protein interaction networks. The activation of this pathway was highly selective in discriminating *MYCN*-amplified neuroblastomas in all three datasets. Our data also suggest that the phosphoinositide 3-kinase (PI3K) inhibitors may provide new opportunities for the treatment of the *MYCN*-amplified neuroblastoma subtype.

## INTRODUCTION

Neuroblastoma arises from sympathetic nervous system embryonal crest cells localized in sympathetic ganglia and adrenal medulla. Approximately 60% of the cases occur in infants less than 2 years old. Neuroblastoma constitutes ~ 6% of all pediatric cancers [1], and causes ~10% of tumor-associated deaths [2]. Most primary tumor sites are within the abdomen, including adrenal medulla and abdominal paraspinal ganglia, while metastases occur in many locations within the body including pelvis, liver, lymph nodes, bone marrow, brain and orbits [3]. The individuals with more differentiated tumor cells have generally better long-time survival prognosis. In contrast, crest-like tumors show less optimistic outcomes. Infants with localized neoplasms have shown the best survival rate and are capable of spontaneous regression [4] (these notable cases are frequently classified as ‘4S’ stage) while older children have higher chances of severe complications and metastases [4]. Striking heterogeneity of the neuroblastoma is connected with impaired neural crest maturation, which involves complex epigenetic reprogramming and alteration of transcriptional factor repertoire [5]. Despite recent survival rate improvements [6, 7], treatment of high-risk and late-stage neuroblastoma cases is still challenging due to the heterogeneity of the disease [8] and existence of treatment-resistant subgroups associated with high lethality [9]. Numerous clinical features such as age, chromosomal abnormalities, histopathological features, tumor ploidy and *MYCN*-amplification are used to assess the risk group and prognosis. In many lower-stage or low-risk cases, only surgery is deemed sufficient. However, in high-risk cases intense chemo- and radio therapies followed by surgery are used [10], strongly contributing to treatment-related morbidity [11]. MYCN-amplified cases are generally stratified into high-risk subgroup and are treated with multimodal cancer therapy including chemotherapy, surgery, radiotherapy, cis-retinoic acid and immunotherapy. Recently, ~60% four-year event-free survival rate has been reported for such patients [5]. Hence, novel target treatment strategies are required to reduce toxicity and improve effectiveness of unfavorable subtype and late-stage NBL therapy.

The *MYCN* amplification arises in 20% of all neuroblastoma cases [12] and in ~50% of the cases associated with poor prognosis and survival [13]. The *MYCN* gene is a member of the *MYC* family of proto-oncogenic transcription factors. It encodes a protein with a basic helix-loop-helix (bHLH) domain. To execute its molecular function, N-Myc protein must dimerize with another bHLH protein in order to bind DNA. Normally, N-Myc protein is expressed in the fetal brain and regulates its development [14]. The attempts to classify molecular data linked with the *MYCN* amplification and/or overexpression have been reported previously, including building molecular networks for few involved proteins [15, 16]. In addition, specific gene expression signatures were proposed to stratify *MYCN*-amplified neuroblastoma patients with respect to poor or optimistic prognosis [17]. However, so far, no integrative large-scale analysis has been published for the gene expression features specific for the *MYCN* amplification in neuroblastoma.

In this report, a multi-level multi-platform transcriptomic data analysis was performed in order to capture a set of molecular characteristics associated with *MYCN* amplification. We found 109 high quality (AUC>0.8) gene expression and 25 molecular pathway activation biomarkers of the *MYCN* amplification. We generated and validated a new molecular pathway crosslinking the identified gene expression biomarkers, termed “*MYCN amplification pathway*” including 41 marker gene products and 23 additional members. Among the marker pathways, the phosphatidylinositol 3’ kinase (PI3K) family members were highly enriched. We propose that in the future the selective inhibitors of PI3K can be used to supplement the current therapies for the *MYCN-*amplified neuroblastomas.

## RESULTS

### Design of the study

Here, we performed a multi-level multi-platform transcriptomic data analysis in order to capture a set of molecular characteristics associated with the MYCN amplification. We compared the mRNA expression data obtained for the neuroblastoma samples with known status of *MYCN* amplification in the three different studies, using the three alternative microarray platforms. For the first two studies (TARGET, n = 243 [18] and MAQCII, n = 477 [19]), the expression data were taken from the open databases. The gene expression was profiled using the Affymetrix HumanExon ST and the Agilent Custom Neuroblastoma Chip microarray platforms, respectively. For each dataset, we identified the expression markers statistically significantly discriminating the *MYCN* amplified group of samples. The marker genes were then intersected and validated in an independent assay. Based on the marker genes coincided in both studies and using the OncoFinder molecular interactions network, we created a new molecular pathway specific to *MYCN* amplification. To validate this pathway, we collected 41 unrelated primary neuroblastoma clinical samples with known status of *MYCN* amplification and profiled gene expression using an alternative customized microarray platform (CustomArray, USA). We took the *pathway activation strength* (PAS) as the *MYCN* pathway biomarker calculated according to the OncoFinder method [20]. The quality of a *MYCN* pathway and of all other biomarkers was tested by calculating the “area-under-curve” (AUC) values [21]. The AUC value is the universal characteristics of robustness and it depends on the sensitivity and specificity of a biomarker. It correlates positively with the biomarker quality and may vary in an interval from 0.5 till 1. The AUC threshold for discriminating good and poor biomarkers is typically 0.7 or 0.75. The items having greater AUC score are considered good-quality markers and vice-versa [22].

### Literature data analysis – TARGET project gene expression data

We extracted gene expression data obtained during the TARGET research project for the 247 neuroblastoma tissue samples, among them 68 had and 175 did not have *MYCN* amplification, for four additional samples the amplification status was unknown [18]. The data included expression levels measured for 22985 known human genes using the Affymetrix HumanExon ST microarray platform. For further investigation, we took the 243 samples with the established *MYCN* amplification status. We used two-level analysis at the gene expression and the pathway activation levels. Molecular pathways regulate all major biological processes in the cell [23–25]. Changes in their activity reflect various differential conditions including malignization of normal human tissues [26, 27]. To measure pathway activities, we calculated the pathway activation strengths (PAS) values. Based on the gene expression profiles, it determines if the pathway is significantly up- or down-regulated compared to the reference, and provides a quantitative measure of this bias. Negative and positive overall PAS values correspond to an inhibited or activated state of a pathway [20]. To calculate the PAS values for the 338 intracellular signaling pathways, we used the OncoFinder protocol [20] because it enables significant reduction of noise for most of the experimental platforms [28].

**Figure 1:**
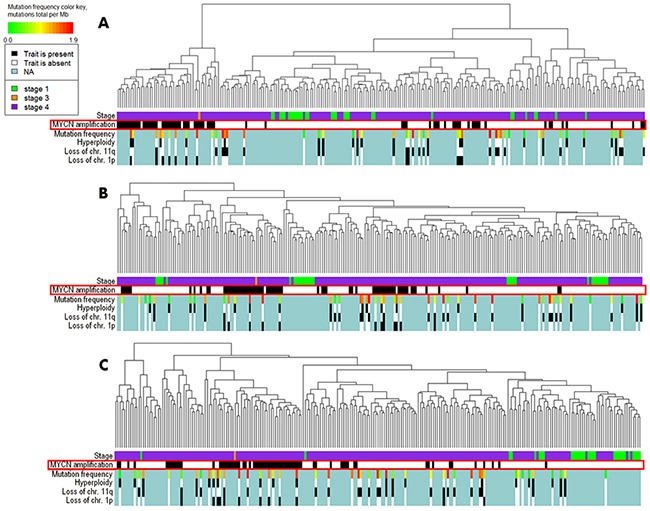
Hierarchical clustering of the TARGET project gene expression data at the levels of pathway activation strength **(A)**, expression of all available genes **(B)** and expression of the available genes involved in the OncoFinder molecular pathway database **(C)**. Clinical and diagnostic features such as the tumor stage, *MYCN* amplification status, mutation frequency, hyperploidy, loss of chromosomal 11q and 1p arms are shown where available on the corresponding marker bars.

We next performed hierarchical clustering analysis (Figure 1). The clustering based on the pathway activation strength (PAS) levels (Figure 1A) displayed a more dense distribution of the *MYCN* –amplified biosamples compared to the clustering based the total gene expression levels (Figure 1B). We next took for clustering only those genes included in the signaling pathways (Figure 1C), and in the latter case the clustering in general reflected the figure seen for the pathway level. At the levels of molecular pathway activation and expression of the pathway genes, the *MYCN* –amplified samples tended to co-clusterize together (Figure 1A and 1C), whereas for the clustering based on the whole set of genes, the MYCN –amplified samples did not form a compact group (Figure 1B).

We next performed a statistical analysis to establish which gene expression profiles and molecular pathway activation strengths may serve as the good quality biomarkers for the discrimination of the *MYCN* –amplified neuroblastomas. With the threshold of AUC 0.8, we identified 175 individual gene expression markers (Supplementary Dataset 1) and 29 pathway activation markers (Supplementary Dataset 2).

### Literature data analysis – MAQC II project gene expression data

**Figure 2:**
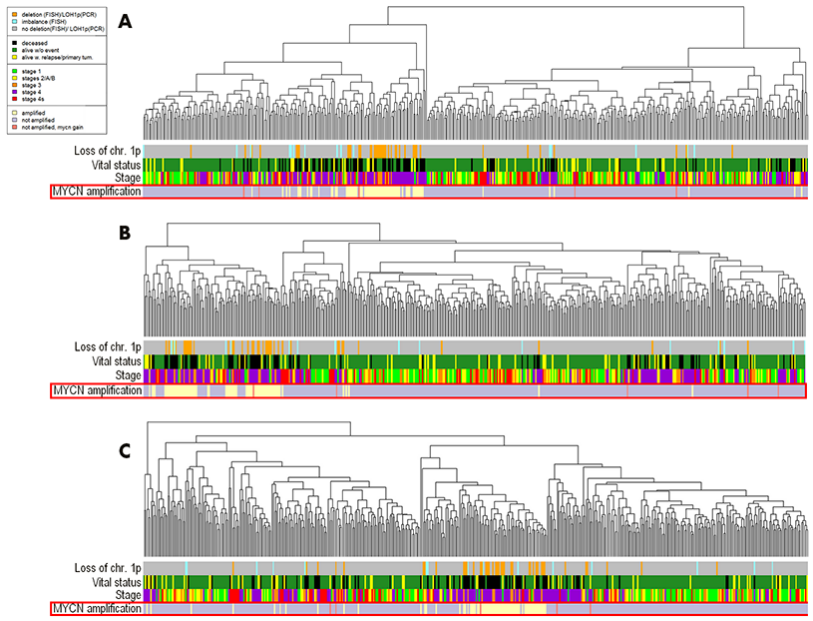
Hierarchical clustering of the MAQC II project gene expression data at the levels of pathway activation strength **(A)**, expression of all available genes **(B)** and expression of the available genes involved in the OncoFinder molecular pathway database **(C)**. Clinical and diagnostic features such as the tumor stage, *MYCN* amplification status, mutation frequency, loss of chromosomal 1p arm, vital status of the patients, are shown where available on the corresponding marker bars.

In the neuroblastoma gene expression dataset linked with the MAQC II research project, the data for 478 tissue samples were present, among them 69 had and 408 did not have *MYCN* amplification, and for one sample the amplification status was unknown [19]. The data included expression profiles for 17360 genes measured using the Agilent Custom Neuroblastoma Chip microarray platform. For further investigation, we took the 477 samples with the established *MYCN* amplification status. We next performed hierarchical clustering analysis at the gene expression and pathway activation strength (PAS) levels (Figure 2). Here, the tight clustering was seen for the *MYCN*-amplified biosamples at all the levels: PAS (Figure 2A), total gene expression (Figure 2B), and expression of genes included in the signaling pathways (Figure 2C). With the threshold of AUC 0.8, we identified 446 individual gene expression markers (Supplementary Dataset 1) and 90 pathway activation markers (Supplementary Dataset 2) of the *MYCN*-amplified fraction of biosamples.

### Intersection of TARGET and MAQC II data

**Figure 3:**
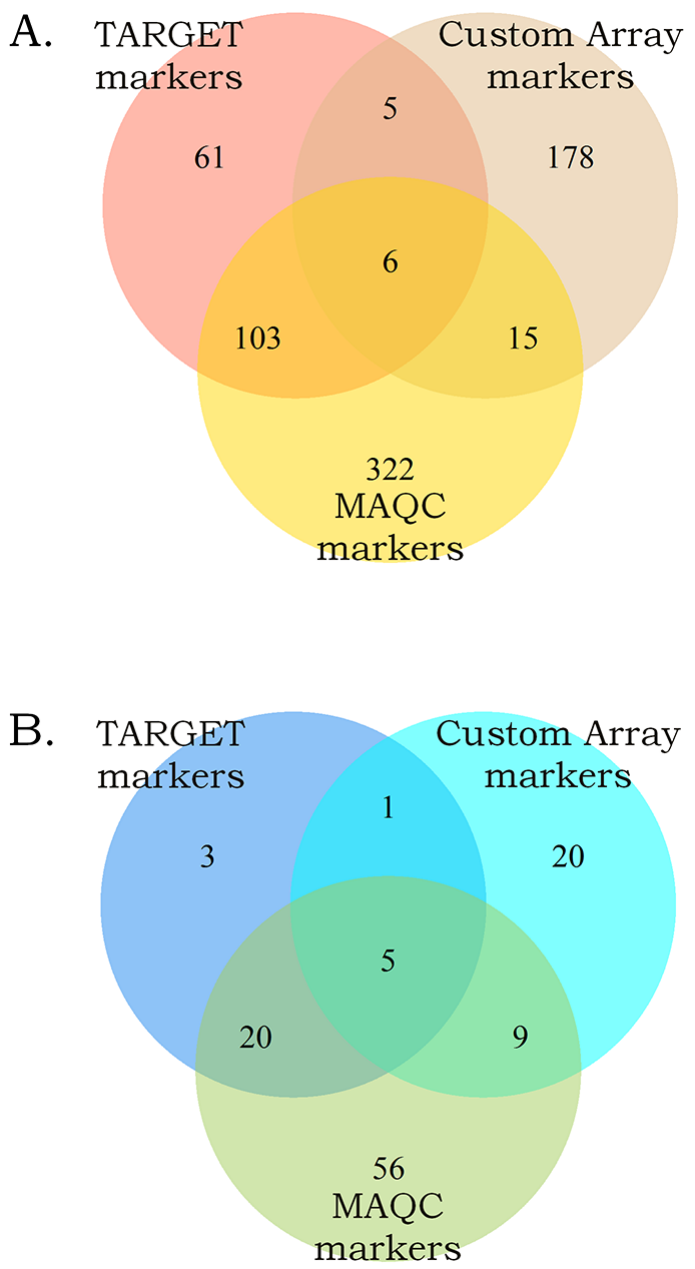
Intersection of the gene expression **(A)** and the pathway activation **(B)** markers of the *MYCN* amplification identified independently in the MAQC, TARGET projects and in this study.

**Figure 4:**
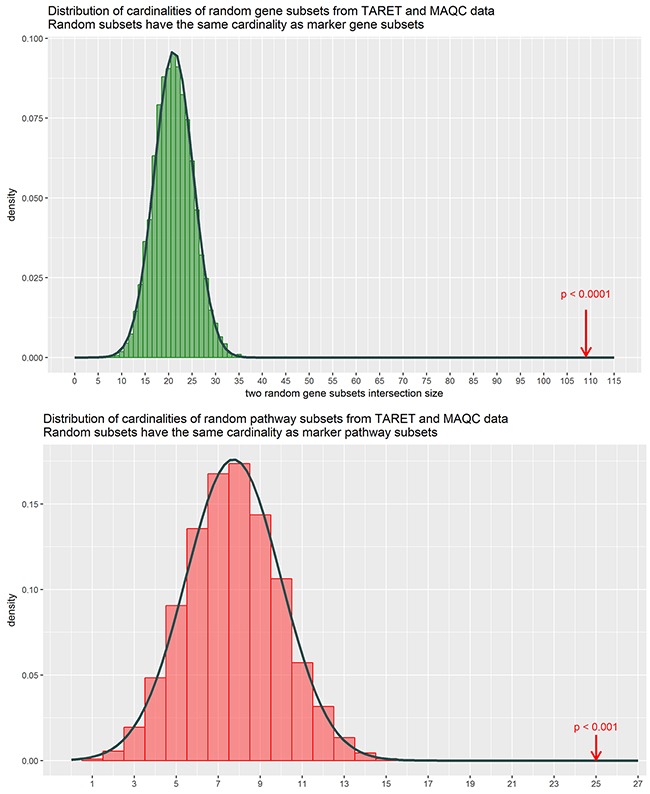
Distributions of cardinalities of random gene samplings from the MAQC and TARGET datasets The cardinalities were obtained by randomly subsetting genes (green) and patways (red) based on the TARGET and the MAQC data with the cardinalities matching that of the obtained marker subsets, and then intersecting them totally 10000 times. Cardinality of the intersection is shown on the horizontal axis. Arrows denote the true marker subset cardinalities, which lie outside of empirical distributions suggesting that these double discriminating genes / pathways are not merely a random noise.

To identify biomarkers common for the two datasets, we intersected the high-quality gene expression and pathway activation markers identified at the previous steps (Figure 3). Figure 3A and 3B show gene expression and pathway activation markers, respectively. Among those, we found 109 gene expression (including *MYCN* gene itself; Supplementary Dataset 3) and 25 pathway biomarkers (Table 1). Both the double gene expression and pathway activation markers showed the appearance distinct from the random subset intersections distribution model for the genes and pathways with high statistical scores (p < 0.0001 and p < 0.001, respectively, Figure 4). This evidences that the double biomarkers intersected not randomly, but due to the true link with the *MYCN* gene amplification features.

**Table 1:** Double pathway activation strength markers identified using literature data with the corresponding ROC-AUC values

Pathway name	AUC, TARGET	AUC, MAQC
ATM_Pathway_Apoptosis_and_Senescence	0.819	0.896
ATM_Pathway_Repair_and_Recombination	0.808	0.854
ATM_Pathway_S-phase_progression	0.836	0.851
BRCA1_Pathway_Base_Excission_Repair	0.83	0.923
BRCA1_Pathway_Cell_Cycle_Arrest_DNA_Repair_Genes_p21_WAF_CIP1_14-3-3_GADD45	0.832	0.927
cAMP_Pathway_Cell_Growth	0.803	0.832
cAMP_Pathway_Cell_Survival	0.839	0.912
cAMP_Pathway_Metabolic_Energy	0.803	0.815
Cellular_Apoptosis_Pathway_Depolarization	0.892	0.855
Cellular_Apoptosis_Pathway_Gene_Expression_BAX_BID_BAK_Ras_Noxa_PUMA_APAF1_Survivin_BCL2_via_TP53	0.862	0.888
Chemokine_Pathway	0.805	0.896
Estrogen_Pathway_Anti-Apoptosis	0.863	0.832
GPCR_Pathway_Gene_Expression_via_JUN_NFKB2_ELK1_SRF_FOS_CREB3	0.812	0.856
Growth_Hormone_Signaling_Pathway_Gene_Expression_via_SRF_ELK1_STAT5B_CEBPD_STAT1_STAT3	0.805	0.868
IL-10_Pathway_IL-10_Responsive_Genes_Transcription_of_BCLXL_Cyclin-D1_D2_D3_Pim1_c-Myc_and_P19(INK4D)_via_STAT3	0.806	0.832
IL-2_Pathway_Apoptosis_Inhibition	0.805	0.869
IP3_Pathway	0.818	0.931
MAPK_Signaling_Pathway_Gene_Expression_Apoptosis_Inflammation_Tumorigenesis_via_MYC_HSF1_STAT2	0.85	0.859
Mitochondrial_Apoptosis_Pathway_Depolarization	0.806	0.895
NGF_Pathway_Actin_Polymerization_Neurite_Outgrowth_and_Differentiation	0.834	0.955
NGF_Pathway_Neurite_Outgrowth_and_Differentiation	0.865	0.948
p53_Signaling_Pathway_Inhibition_of_IGF1R_mTOR_Pathways	0.884	0.919
PPAR_Pathway	0.802	0.83
Ras_Pathway_Cell-Cell_Junctions	0.81	0.835
WNT_Pathway_Cell_Survival	0.823	0.92

The bioinformatic Gene Ontology (GO) enrichment analysis showed that the double marker genes formed six completely statistically significant functional clusters and many distinct significantly enriched GO terms (Supplementary Dataset 4), featuring purine nucleotide biosynthesis, tetrahydrofolate and one-carbon metabolism, building mitochondrial matrix, biosynthesis of amino acids, tRNA aminoacylation for protein translation, NAD(P)-linked oxidation-reduction processes, ATP binding, tyrosine phosphatase activity, p53 signaling, cell cycle progression, G1/S and G2/M checkpoints.

**Figure 5:**
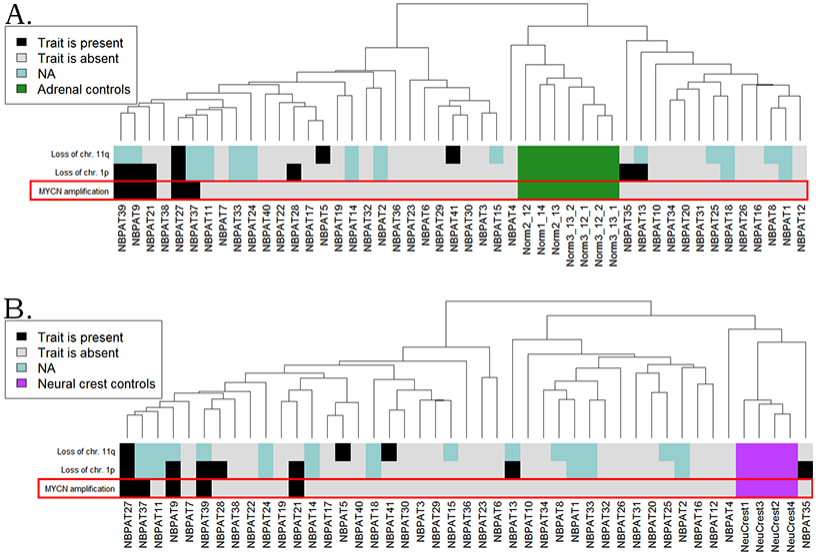
Hierarchical clustering of the CustomArray experimental data at the level of pathway activation strength, normalized on the normal adrenal gland **(A)** and embryonic neural crest **(B)** controls. The diagnostic features such as *MYCN* amplification status, loss of chromosomal 1p and 11p arms, are shown on the corresponding marker bars.

To generate a molecular pathway crosslinking the identified gene expression biomarkers, we used the OncoFinder pathway creator module. Based on the available knowledge on protein-protein interactions, it enables creating a network linking maximum number of featured gene products with a minimal number of intermediary products involved. We generated a new pathway termed “*MYCN* pathway” including 41 double marker gene products and 23 intermediary members (Figure 5; gene products listed on Supplementary Dataset 5). Totally, we identified 25 double marker molecular pathways (Table 1). Of them, two pathways (8%) are clearly connected with the activity of C-MYC, a well-known oncogenic transcriptional factor closely related to N-MYC. Those pathways are: *IL-10_Pathway_ Transcription_of_BCLXL_Cyclin-D1_D2_D3_Pim1_c-Myc_and_P19(INK4D)_via_STAT3* and *MAPK_Signaling_Pathway_Gene_Expression_Apoptosis_Inflammation_Tumorigenesis_via_MYC_HSF1_STAT2*. The first pathway deals with the IL10 influence on C-MYC expression, whereas the second, in turn, represents C-MYC influence on the expression of its responsive genes. The other marker pathways were linked with the activities of ATM kinase (3 pathways) and BRCA1-dependent DNA repair (2 pathways), with apoptosis and p53 signaling (4 pathways), PPAR signaling (1 pathway), chemokine and GPCR signaling (2 pathways), branches of NGF pathway regulating cytoskeleton (2 pathways), one Ras signaling pathway regulating intercellular interactions, four cell survival pathways via cAMP, Estrogen, IL2 and Wnt signaling, and four cell growth-promoting pathways via cAMP, IP3 and growth hormone signaling (Table 1). Among those, nine pathways (36%), namely, ATM-, BRCA1- and p53/apoptosis signaling pathways exactly matched the marker activities previously identified at the individual gene level using the GO analysis (Supplementary Dataset 4).

We next compared the gene contents of the 25 double marker pathways and found that some genes were highly enriched among them (Table 2). For example, twelve phosphoinositide 3-kinase (PI3K) family members simultaneously appeared in 9/25 (36%) of the marker pathways. The *TP53* gene participated in seven featured pathways, whereas four guanine nucleotide-binding protein genes, four *MAPK*-family members, *ATM* and *AKT1* genes each took part in five marker pathways.

**Table 2:** Gene enrichment statistics for the double marker molecular pathways

Gene name	Occurrence
*PIK3C2A, PIK3C2B, PIK3C2G, PIK3C3, PIK3CA, PIK3CB, PIK3CD, PIK3R1, PIK3R2, PIK3R3, PIK3R4, PIK3R5*	9
*TP53*	7
*GNAS*	6
*AKT1*	5
*ATM*	5
*GNA11, GNA12, GNA13, GNA14, GNA15, GNAQ, GNG2, GNG3, GNG4*	5
*MAPK1, MAPK3, MAPK8, MAPK9, MAPK10, MAPK12*	5
*ADCY1, ADCY2, ADCY3*	4
*CHEK2*	4
*GNAL, GNAO1, GNAT1, GNAZ, GNB1, GNB2, GNB3, GNB4, GNB5, GNG5, GNG7, GNG8, GNG10, GNG11, GNG12, GNG13, GNGT1, GNGT2*	4
*HRAS*	4
*MAP3K1, MAPK11, MAPK13, MAPK14*	4
*PLCG1, PLCG2*	4
*PRKACA, PRKACB, PRKACG*	4
*STAT3, STAT5A, STAT5B*	4

### Experimental validation of *MYCN* biomarkers

To validate the identified *MYCN*-specific gene expression pathway, we collected 41 unrelated neuroblastoma clinical samples, including 5 *MYC*–amplified and 36 wild type tissue blocks. The samples were formalin-fixed, paraffin embedded tissue blocks corresponding to surgically resected neuroblastoma tissues. The mean age of the patients was 24 months (Supplementary Dataset 6). To normalize gene expression profiles, we took seven adrenal biopsy samples isolated from the adult donors. Alternatively, we used another set of normal tissues isolated from the biosamples of neural crest tissue from four embryonal donors. From all the tissue samples, we extracted total RNA and generated gene expression libraries for microarray hybridization. We used customized electrochemical microchip platform manufactured by CustomArray (USA) enabling direct oligonucleotide synthesis on the array [29]. We produced the arrays with 6020 oligonucleotide probes corresponding to 3706 human genes involved in 378 intracellular signaling pathways, as described previously [30]. Following library preparations and hybridizations, the microarray hybridization signals were quantile normalized, and the gene expression data were deposited in Gene Expression Omnibus (GEO) database with the accession number GSE96631. Neuroblastoma transcriptional profiles were analyzed in three ways by comparing with (*i*) the normal adrenal, (*ii*) normal neural crest tissue samples, and (*iii*) with the mean gene expression levels calculated for all the neuroblastoma samples. Similar to the TARGET and MAQC II datasets, the *MYCN-*amplified biosamples clustered together on the dendrograms for both the healthy adrenal (Figure 5A) and embryonal neural crest (Figure 5B) controls.

To assess quality of the *MYCN amplification pathway* in discriminating the experimental *MYCN* -amplified and wild type neuroblastoma samples, we calculated this pathway activation strength (PAS) according to the OncoFinder method [20]. We next modeled the use of the PAS scores as the biomarkers of *MYCN* amplification. For the (*i-iii*) normalizations, the*MYCN amplification pathway* showed a good performance with the AUC scores of 0.778, 0.767 and 0.711, respectively (Table 3).

**Table 3:** Validation of the MYCN amplification pathway on the experimental and literature datasets

Dataset	AUC (*MYCN pathway*)
Custom Array / mean transcriptome	0.711
Custom Array / healthy adrenal glands	0.778
Custom Array / embryonal neural crest cells	0.767
TARGET / mean transcriptome	0.791
MAQC / mean transcriptome	0.861

The major Gene Ontology terms statistically significantly linked with the *MYCN amplification pathway* were: G2/M checkpoint regulation and DNA damage response, p53 signaling pathway, cell cycle progression, maintaining dopaminergic/adrenergic/serotoninergic/gluta-matergic synapses, phospholipid signaling, gene signatures associated with prostate, pancreatic, thyroid, renal, bladder, non-small cell lung cancers, acute myeloid and chronic myeloid leukemia, melanoma, glioma, central carbon metabolism in cancer, regulation of PI3K signaling, MAPK signaling, Ras signaling, tyrosine phosphatase activity, and many other molecular processes (Supplementary Dataset 7).

**Figure 6:**
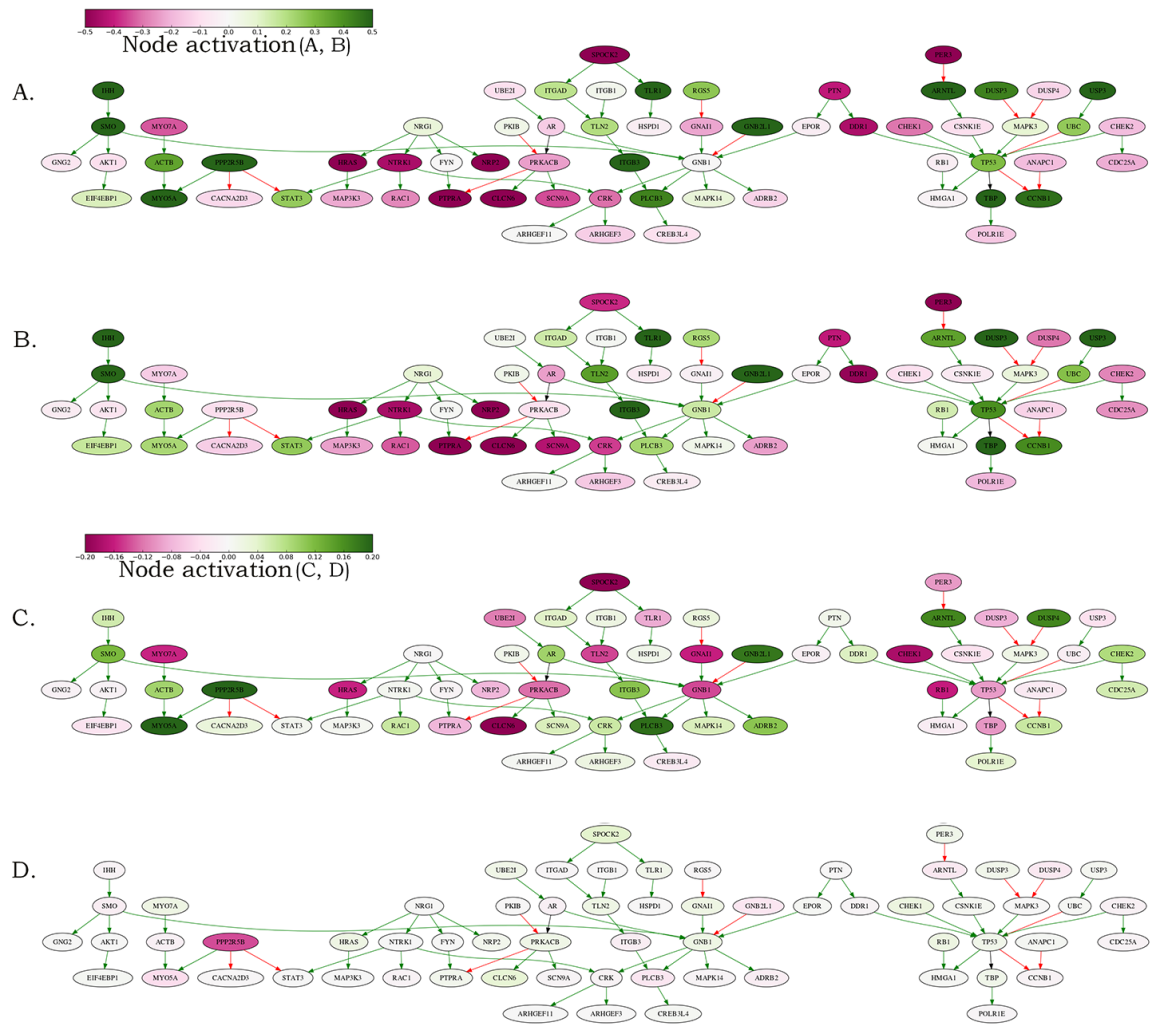
Schematic representation of the *MYCN* amplification molecular pathway The pathway activation features are shown for the averaged *MYCN*-amplified and non-amplified transcriptomes normalized on the normal adrenal gland controls and on the averaged neuroblastoma transcriptional profile. **(A)** *MYCN*-amplified, adrenal-normalized. **(B)** Non-amplified, adrenal-normalized. **(C)** *MYCN*-amplified, neuroblastoma-normalized. **(D)** Non-amplified, neuroblastoma-normalized. The pathway is shown as an interacting network, green arrows indicate stimulation, red arrows – inhibition. Color bars represent activations of the corresponding pathway nodes and correspond to the logarithms of the case-to-normal (CNR) expression rate for each node.

Taken together, this suggests that the established *MYCN amplification pathway* may be used as a good-quality biomarker for the discrimination between the *MYCN-*amplified and wild-type neuroblastomas. When normalized on the normal tissues (*i-ii*), the expression features of the *MYCN amplification pathway* nodes were usually regulated in the same direction in both the *MYCN-*amplified and wild-type neuroblastomas (Figure 6A and 6B). However, they showed bigger difference when normalized on the mean neuroblastoma expression level (*iii*), Figure 6C and 6D. This suggests that the *MYCN* amplification in neuroblastoma is linked with the up- or down regulation of many genes. The extent of their transcription levels can distinguish between the *MYCN* amplified and non-amplified neuroblastomas (Figure 6).

## DISCUSSION

In this study, we tried to identify a sustainable gene expression pattern associated with the amplification of a protooncogene *MYCN,* and molecular processes associated therewith. We used two published high throughput gene expression datasets obtained using two alternative microarray platforms: Agilent Custom Human Neuroblastoma Chip and Affymetrix Human Exon ST array. To identify molecular profiles associated with the *MYCN* amplification, we analyzed both gene expression and molecular pathway activation features. We intersected the good-quality biomarkers (AUC>0.8) identified for these two datasets, and identified double biomarkers at the gene expression (n=109) and the pathway activation (n=25) levels. For the gene expression markers, we performed a search of the enriched Gene Ontology (GO) terms and found statistically significant enrichment in the processes of purine nucleotide biosynthesis, ATP-binding, tetrahydrofolate metabolism, building mitochondrial matrix, biosynthesis of amino acids, tRNA aminoacylation for protein translation, and NADP-linked oxidation-reduction processes. To combine the identified marker gene products to a single regulatory network, we created a new molecular pathway termed “*MYCN amplification pathway*” including 41/109 double marker gene products and 23 intermediary members. We validated this pathway on the additional sampling of 5 *MYCN*-amplified and 36 non-amplified neuroblastoma samples, experimentally profiled here using an alternative microarray platform (Table 3). Using three alternative experimental normalization methods, the *MYCN amplification pathway* was confirmed to be a high-quality biomarker of the *MYCN* amplification. We therefore suggest that this novel molecular pathway built using purely agnostic high-throughput analytic approaches may be recruited to analyze in depth the molecular consequences of *MYCN* amplifications in neuroblastoma in further investigations. Alternatively, it may be used to assess molecular phenotypes of the individual neuroblastomas.

At the same time, we found 25 intersected double marker pathways. Among them, 2 pathways were connected with the activity of C-MYC, known oncogenic transcriptional factor closely related to N-MYC. The other marker pathways were connected with the ATM kinase (3 pathways) and BRCA1-dependent DNA repair (2 pathways), with apoptosis and p53 signaling (4 pathways), PPAR signaling (1 pathway), chemokine and GPCR signaling (2 pathways), branches of NGF pathway regulating cytoskeleton (2 pathways), one Ras signaling pathway regulating intercellular interactions, four cell survival pathways via cAMP, estrogen, IL2 and Wnt signaling, and four cell growth-promoting pathways via cAMP, IP_3_ and growth hormone signaling (Table 1). Importantly, among them, nine pathways (36%), namely, ATM-, BRCA1- and p53/apoptosis-related signaling pathways matched the marker activities identified at the gene expression level using the Gene Ontology terms enrichment analysis in this study.

Many of those pathways were previously published in relation with the severity of neuroblastoma, such as AKT signaling [31], cAMP- and IP_3_–dependent signal transduction mechanisms [32, 33], and also estrogen [34], growth hormone signaling regulating glucose uptake [35], MAPK [36], PPAR [37], NGF [38] and Ras [36] signaling.

Among the members of the twenty-five double marker pathways specifically regulated in the *MYCN-*amplified tumors, we found that some genes were highly enriched. For example, twelve phosphoinositide 3-kinase (PI3K) family members simultaneously occurred in 9/25 (36%) of the marker pathways. The *TP53* gene occurred in the seven featured pathways, and four *MAPK* family members, *ATM* and *AKT1* genes each took part in five marker pathways. This may suggest that the activity of phosphoinositide 3-kinases may be central in translating the molecular effects of *MYCN* amplifications to cell cycle progression. Indeed, the PI3K proteins promote cell growth and survival by activating Akt signaling. In turn, Akt activation has been previously reported a strong prognostic indicator of decreased survival in neuroblastoma [39]. In addition, Akt activation correlates with more aggressive disease, including *MYCN* amplification, advanced stage and unfavorable histological features. The Akt activation in neuroblastoma proceeds in a PI3K-dependent manner, because the PI3K inhibitor LY294002 completely reversed the effects of the insulin-like growth factor–mediated activation of Akt and its protection of neuroblastoma cells from the apoptosis [39]. Another line of evidence suggests that the inhibition of PI3K/Akt signaling by an Akt-specific inhibitor perifosine improves progression-free survival in the patients with high-risk neuroblastomas [40]. We propose, therefore, that the available PI3K inhibitors, such as the LY294002, Idelalisib and Quercetin, may be used to supplement the current therapies for the *MYCN-*amplified neuroblastomas. These data shed light on a variety of molecular processes orchestrated by the amplification of *MYCN* gene and may help developing better anti-cancer molecular therapies in the future.

## MATERIALS AND METHODS

### Biosamples

For this study, we used forty-one experimental formalin-fixed, paraffin-embedded (FFPE) neuroblastoma tissue samples obtained from 41 patients treated at the D. Rogachev Center of Pediatric Hematology, Oncology and Immunology (CPHOI), Moscow. 7 tissue samples for adrenal non-cancer controls were collected at the Department of Pathology at the Faculty of Medicine, Moscow State University, from autopsies taken from 3 independent adult healthy donors killed in road accidents. The 4 embryonal normal neural crest biosamples were taken from the post-mortal prenatal unrelated human donors. For all the biosamples, informed written consents to participate in this study were collected from the patient's representatives. The consent procedure and the design of the study were approved by the ethical committees of the CPHOI, of the First Oncology and Advisory Center, Moscow, and of the Engelhardt Institute of Molecular Biology. Both the tumors and normal tissues were evaluated by a pathologist to confirm the diagnosis and estimate the tumor cell numbers.

### Synthesis of microarrays

B3 microarray synthesizer (CustomArray, USA) was used for forty nucleotides-long oligonucleotide probe synthesis on CustomArray ECD 4×2K/12K slides. Synthesis was performed according to the manufacturer's recommendations. Three replicates of total 6020 unique oligonucleotide probes specific to 3706 human gene transcripts were placed on each chip. Chip design was performed using Layout Designer software (CustomArray, USA). For the custom microchip, we used original oligonucleotide probe sequences of the Illumina HT 12 v4 platform.

### Library preparation and hybridization

Complete Whole Transcriptome Amplification WTA2 Kit (Sigma) was used for reverse transcription and library amplification. Manufacturers protocol was modified by adding to amplification reaction dNTP mix containing biotinylated dUTP, resulting to final proportion dTTP/biotin-dUTP as 5/1. Microarray hybridization was performed according to the CustomArray ElectraSense™ Hybridization and Detection protocol. Hybridization mix contained 2.5 ug of labeled DNA library, 6X SSPE, 0.05% Tween-20, 20mM EDTA, 5x Denhardt solution, 100 ng/ul sonicated calf thymus gDNA, 0,05% SDS. Hybridization mix was incubated with chip overnight at 50°C. Hybridization efficiency was detected electrochemically using CustomArray ElectraSense™ Detection Kit and ElectraSense™ 4×2K/12K Reader.

### Initial processing of microarray data

Probe signals were geometrically averaged, thus obtaining expression value for each specific type of the probe. Then quantile normalization [41] was performed using the ‘preprocessCore’ R package [42], and 3706 genes corresponding to the experimental custom array design were selected for further analysis. Gene expression data were deposited in Gene Expression Omnibus database with the accession number GSE96631.

Open access microarray data containing gene expression profiles for the high-risk neuroblastoma samples, and the available clinical information, were obtained from the TARGET (Therapeutically Applicable Research To Generate Effective Treatments) project website. The data on mutation rate and chromosomal abnormalities for 48 microarray-profiled TARGET patients were obtained from [18].

The MAQCII (ArrayExpress) gene expression data were extracted from the dataset tagged with the ID ‘E-MTAB-179’. The data preparation and quantile normalization were made by the authors according to their protocol ( http://www.ebi.ac.uk/arrayexpress/experiments/E-MTAB-179/protocols/), and we performed no pre-processing except for omitting probes with the negative expression values to counter the effect of inaccurate measurements of strongly underexpressed genes.

### Functional annotation of gene expression

The SABiosciences signaling pathways knowledge base was used to determine structures of intracellular pathways, as described previously [43]. We applied the original OncoFinder algorithm [20] for functional annotation of the primary expression data and for calculating pathway activation strength (PAS) scores and cancer-to-normal ratios (CNRs). CNRn is the ratio of the expression levels of a gene n in the sample under investigation to the average expression in the control group of samples. In this study, the PAS scores were obtained according to [20]. PAS can take both positive and negative values meaning over- or underactivation relative to control tissue. Results for the 378 molecular pathways obtained for each sample are shown on Supplementary Dataset 8). The MAQC II and TARGET data were normalized on the average gene expression profiles for the respective neuroblastoma datasets. The experimental gene expression levels were normalized either on the normal adrenal or neural crest tissues, or on the average gene expression profile for the experimental neuroblastoma dataset.

### Statistical analysis

Hierarchical clustering heatmaps and dendrograms with Euclidean distance and complete-linkage were generated using heatmap.2 function from “gplots” package [44]. Pathways which returned the same PAS scores for all the samples were removed from the analyses during calculations related to biomarker detection. The AUC (area under curve) values were calculated using ‘caTools’ R package [45] for each dataset to estimate how efficient each gene/pathway is in separating *MYCN*-amplified patients from the patients without amplification. The genes contained in OncoFinder signaling pathway knowledgebase were left in each dataset for consistency. Then genes/pathways exceeding the AUC threshold were filtered and intersected for the datasets comparison.

To test the significances of the intersection results (e.g. what is the likelihood to randomly obtain such number of markers in total intersection), a simulation was performed, randomly selecting gene / pathway subsets and intersecting them. The histograms for the resulting distributions for genes and pathways were drawn using ‘ggplot2’ package [46]. The resulting discrete distributions were approximated by normal distributions with parameters estimated from simulated data to test for the result's significance.

### Gene enrichment analysis

To perform the Gene Ontology (GO) database investigations for the gene sets associated with the discovered biomarkers, we performed enrichment analysis using DAVID functional annotation clustering software [47]. We used ‘medium’ default clustering parameters. The gene symbols corresponding to the respective array platforms were transformed to Entrez gene using biomaRt package [48] and used as the background for the respective calculations for the selected gene sets. Terms that had 50% or more common members were iteratively merged to obtain final clusters.

### Pathway reconstruction

A MYCN amplification pathway was reconstructed using the established pairwise molecular interactions signatures extracted from the Biocarta, KEGG, NCI, Quiagen and Reactome databases (https://cgap.nci.nih.gov/Pathways/BioCarta_Pathways, http://www.genome.jp/kegg/pathway.html, http://www.ndexbio.org/#/user/301a91c6-a37b-11e4-bda0-000c29202374, https://www.qiagen.com/at/shop/genes-and-pathways/pathway-central/?akamai-feo=off&f=po%3a2, http://www.reactome.org/pages/download-data/). We first used Dijkstra algorithm to detect shortest pairwise distances between the gene products on the graph constructed by merging all the databases, and then cut loosely-connected branches.

## SUPPLEMENTARY MATERIALS DATASET


















