# Supplementary material for: Gene expression and molecular pathway activation signatures of MYCN-amplified neuroblastomas
Source: Oncotarget. 2017 Jul 28;8(48):83768–80. doi: 10.18632/oncotarget.19662 (PMC5663553; doi:10.18632/oncotarget.19662)
Supplement: Supplementary file 1 [file oncotarget-08-83768-s001.pdf]

Gene expression and molecular pathway activation signatures of *MYCN*-amplified neuroblastomas

SUPPLEMENTARY MATERIALS

Supplementary Dataset 1: TARGET / MAQC project data gene expression markers - Contains gene expression markers for TARGET and MAQC data on separate tabs; contains AUC value and up/down regulation status in amplified and non-amplified patients relative to the mean transcriptome for each marker gene.

See Supplementary File 1

Supplementary Dataset 2: TARGET / MAQC project data pathway activation markers - Contains pathway activation markers for TARGET and MAQC data on separate tabs; contains AUC value and up/down regulation status in amplified and non-amplified patients relative to the mean transcriptome for each marker pathway.

See Supplementary File 2

Supplementary Dataset 3: Intersection of TARGET and MAQC gene markers - Contains gene id and AUC values corresponding to their performance in TARGET and MAQC data.

See Supplementary File 3

Supplementary Dataset 4: DAVID clustering for intersecting gene markers - This table is formatted as standard DAVID functional annotation clustering output file.

See Supplementary File 4

Supplementary Dataset 5: Composition of MYCN amplification pathway - Detailed information on genes contained in newly constructed MYCN amplification pathway.

See Supplementary File 5

Supplementary Dataset 6: Information regarding Custom Array patients - Contains patient ID, MYCN status, 1p / 11q loss status and age at manifestation in months.

See Supplementary File 6

Supplementary Dataset 7: GO terms linked with newly constructed MYCN amplification pathway - The major Gene Ontology terms statistically significantly linked with the MYCN amplification pathway are the following: G2/M checkpoint regulation and DNA damage response, p53 signaling pathway, cell cycle progression, maintaining dopaminergic/adrenergic/serotoninergic/glutamatergic synapses, phospholipid signaling, gene signatures associated with prostate, pancreatic, thyroid, renal, bladder, non-small cell lung cancers, acute myeloid and chronic myeloid leukemia, melanoma, glioma, central carbon metabolism in cancer, regulation of PI3K signaling, MAPK signaling, Ras signaling, tyrosine phosphatase activity.

See Supplementary File 7

Supplementary Dataset 8: Results for the 378 molecular pathways obtained for each sample - Includes all three datasets (TARGET, MAQC, Custom Array with different controls).

See Supplementary File 8
